# ICD-10 based machine learning models outperform the Trauma and Injury Severity Score (TRISS) in survival prediction

**DOI:** 10.1371/journal.pone.0276624

**Published:** 2022-10-27

**Authors:** Zachary Tran, Arjun Verma, Taylor Wurdeman, Sigrid Burruss, Kaushik Mukherjee, Peyman Benharash

**Affiliations:** 1 Cardiovascular Outcomes Research Laboratories (CORELAB), David Geffen School of Medicine, University of California, Los Angeles, California, United States of America; 2 Division of Acute Care Surgery, Department of Surgery, Loma Linda University Medical Center, Loma Linda, California, United States of America; Monash University, AUSTRALIA

## Abstract

**Background:**

Precise models are necessary to estimate mortality risk following traumatic injury to inform clinical decision making or quantify hospital performance. The Trauma and Injury Severity Score (TRISS) has been the historical gold standard in survival prediction but its limitations are well-characterized. The present study used *International Classification of Diseases 10*^*th*^ *Revision* (ICD-10) injury codes with machine learning approaches to develop models whose performance was compared to that of TRISS.

**Methods:**

The 2015–2017 National Trauma Data Bank was used to identify patients following trauma-related admission. Injury codes from ICD-10 were grouped by clinical relevance into 1,495 variables. The TRISS score, which comprises the Injury Severity Score, age, mechanism (blunt vs penetrating) as well as highest 24-hour values for systolic blood pressure (SBP), respiratory rate (RR) and Glasgow Coma Scale (GCS) was calculated for each patient. A base eXtreme gradient boosting model (XGBoost), a machine learning technique, was developed using injury variables as well as age, SBP, RR, mechanism and GCS. Prediction of in-hospital survival and other in-hospital complications were compared between both models using receiver operating characteristic (ROC) and reliability plots. A complete XGBoost model, containing injury variables, vitals, demographic information and comorbidities, was additionally developed.

**Results:**

Of 1,380,740 patients, 1,338,417 (96.9%) survived to discharge. Compared to survivors, those who died were older and had a greater prevalence of penetrating injuries (18.0% vs 9.44%). The base XGBoost model demonstrated a greater receiver-operating characteristic (ROC) than TRISS (0.950 vs 0.907) which persisted across sub-populations and secondary endpoints. Furthermore, it exhibited high calibration across all risk levels (R^2^ = 0.998 vs 0.816). The complete XGBoost model had an exceptional ROC of 0.960.

**Conclusions:**

We report improved performance of machine learning models over TRISS. Our model may improve stratification of injury severity in clinical and quality improvement settings.

## Background

Traumatic injuries account for 8% of global deaths and have far reaching implications in chronic disabilities [1]. Given the wide spectrum of injuries, accurate predictive modeling of mortality in trauma victims is paramount to several clinical and programmatic aims. Such models may be used to support benchmarking efforts, quality improvement research and real-time clinical decision-making [2, 3]. However, currently used trauma scores, such as the Injury Severity Score (ISS), have several significant pitfalls. Initially developed in 1974 for research and quality monitoring purposes, it is reliant on additional administrative coding, was not designed to be a comprehensive summary of all injuries and does not consider in-hospital factors which may be important for adjustment [4–6]. The Trauma and Injury Severity Score (TRISS) mitigated some shortcomings of the ISS by incorporating physiologic variables routinely collected upon arrival to the emergency department [7]. Nonetheless, both models rely on Abbreviated Injury Scale (AIS) data that are not regularly collected in all centers and require dedicated coders.

More recently, models derived from *International Classification of Diseases* (ICD) codes have attempted to address some of the limitations noted in AIS-based risk algorithms. The Trauma Mortality Prediction Model (TMPM), which employs traditional logistic regression, has garnered interest as a feasible alternative [8, 9]. Nonetheless, this methodology fails to account for the complex interplay of injuries and their impact on mortality. Machine learning (ML)-based models, whose strengths lie in complex outcome prediction, may incorporate these relationships through their decision tree architecture [10, 11]. Its prior applications have included predicting complications following shoulder arthroplasty, bleeding following colonic resection, among others [12]. In fact, recent work from our group demonstrated improved discrimination and calibration of eXtreme gradient boosting (XGBoost), a ML approach, in mortality prediction compared to logistic regression, ISS and TMPM [13].

Given that our prior work only incorporated injury variables, our aim was to determine whether inclusion of physiologic factors augment the model’s power in predicting mortality [14, 15]. Although the TRISS score has not been validated for outcomes other than survival, we additionally sought to explore the validity of both ML and TRISS models in a number of in-hospital complications. In the present study, we used ICD-10 injury codes in conjunction with vital signs, Glasgow Coma Scale (GCS), age and mechanism to develop and validate an improved machine learning model. We hypothesized that our model would persistently demonstrate superior performance compared to TRISS and would have high performance in prediction of in-hospital complications.

## Materials and methods

### Data source and study population

Patients of all ages admitted following traumatic injury were identified using the National Trauma Data Bank (NTDB) from October 2015 to December 2017. The NTDB is the largest, voluntarily reported national trauma database in the United States with greater than 10 million aggregate records from nearly 800 participating hospitals. Patients with traumatic mechanisms of injury were identified using ICD-10-CM codes V00-Y99. Those who sustained burn injuries or had admissions from drowning/submersion, environmental or exertional causes (ICD-10-CM: W65-W99, X00-X50) were excluded to enhance patient homogeneity. Patients transferred to another hospital or with missing survival information, were excluded (9.0%: 2.5% transferred out, 6.5% missing survival).

### Study variables and outcomes

The ISS for each patient is submitted by the respective trauma center through AIS coding and quantifies injury severity with a range of 1–75 (ISS). It is calculated as the sum of squares for the highest AIS scores for the three most severely injured body regions. The TRISS score, which comprises the ISS, age, mechanism (blunt vs penetrating) as well as the highest 24-hour values for systolic blood pressure (SBP), respiratory rate (RR) and GCS was calculated for each patient. Patients with missing values for any of the above variables were excluded from further analysis (14.3% patients).

Variables used in the ML models were derived using ICD-10-CM codes, with each patient having a maximum of 50 injury codes. They contain descriptors for “initial encounter”, “subsequent encounter”, and “sequela.” To ensure that only first-time injuries were evaluated, analysis was limited to injury codes that specify “initial encounter.” Codes are compiled at the end of each patient’s hospitalization using documentation from medical examiners and operative reports, radiologic studies as well as physicians’ notes. In the present study, 8,021 ICD-10-CM codes were grouped by clinical relevance into 1,495 final variables, as previously described by our group [13]. Notably, both ISS and ICD-10-CM nomenclature describe “unsurvivable” injuries. Codes and patients that sustained these injuries were retained in our study. To ensure a fair comparison of ML and TRISS, a base ML model was developed to include mechanism of injury, age, SBP, RR and GCS. The full ML model, which contained additional NTDB-provided variables shown in S1 Table, was also developed. A schematic demonstrating variables used in each model is shown in S1 Fig.

The primary outcome of the study was survival to discharge at index hospitalization. Secondary outcomes included in-hospital stroke (ischemic or hemorrhagic stroke), cardiac complications (myocardial infarction, non-traumatic cardiac arrest, ventricular arrhythmia), pneumonia, acute respiratory failure (ARF) (acute respiratory distress syndrome), deep vein thrombosis (DVT), pulmonary embolism (PE), massive transfusion (≥10 units within 24 hours), acute kidney injury (AKI), infection (surgical site infection, line infection, sepsis) and need for intensive care unit (ICU) admission. Outcomes were defined using the NTDB data dictionary and ICD-10-CM codes defined elsewhere [16]. For secondary outcomes, the base ML and TRISS models were compared. Importantly, the TRISS was validated for survival, but not for the secondary outcomes. Analysis was performed in order to provide a reference group with the ML model.

### Statistical analyses

Categorical variables are reported as proportions while continuous variables are reported as medians with interquartile range (IQR). Patient demographics were assessed using the Kruskal-Wallis and the chi-square tests for continuous and categorical variables, respectively. Standard mean differences (SMD) were obtained to adjust for population size. We developed models with the XGBoost algorithm, a machine learning technique in which decision trees are trained in a stage-wise manner [17]. Using errors from previous iterations, models are refined with the development of each subsequent decision tree. This technique of sequential training of decision trees is called gradient boosting. The final output is the average prediction of all individual decision trees. The performance of an XGBoost model can be optimized through tuning of hyperparameters, which are used to control the learning process. Hyperparameter tuning was performed using the RandomizedSearchCV function in Python. This tool randomly searches through a broadly defined hyperparameter space and evaluates models using the cross-validated greatest area under the receiver operating characteristic curve (ROC). The hyperparameters that yield the highest ROC are chosen. In the present study, a negligible impact of hyperparameter tuning was noted; therefore, default values were maintained (S2 Table) [18].

### Model development and training

For all analyses, covariates used are shown in S1 Fig with patients randomly assigned into derivation (50%) and validation (50%) cohorts. Models were evaluated using 10-fold cross-validation for out of sample performance. To assess generalizability across patient cohorts, sensitivity analysis was performed on six subgroups of patients, including those (1) with head injuries, (2) without head injuries, (3) with penetrating or (4) blunt traumatic mechanisms, (5) <50 years old and (6) ≥50 years old. Head injuries were defined as patients who had at least one cranial injury code as previously defined [13].

Model discrimination was compared using the ROC, precision (positive predictive value), recall (sensitivity), specificity and with confusion matrices. Precision-recall curves were constructed to show sensitivity and positive predictive value across all risk-thresholds [19]. Reliability plots were constructed by plotting observed versus expected mortality rates and compared using the coefficient of determination (R^2^). The Brier score was used to measure the accuracy of probabilistic predictions [20]. Finally, SHapley additive values were utilized to enhance the interpretability of our ML model. This method uses game theory principles to estimate the incremental impact of variable value on the output of a decision tree model [21]. The resulting SHAP summary plot generated from these values combines feature importance with feature effects on a model.

To account for a large number of missing values for components of the TRISS score, sensitivity analysis was performed using simple imputation. Medians were used for continuous variables while the mode was used for categorical values. Statistical significance was defined as α<0.05 and SMD>0.1. All analyses were conducted using Stata 16.0 (StataCorp LLC, College Station, TX) and Python 3.8.10 libraries: *pandas 1*.*1*.*5*, *sklearn 0*.*24*.*2*, *xgboost 1*.*6*.*1* and *shap 0*.*40*.*0* [17, 21–23]. This study was deemed exempt from full review by the Institutional Review Board at the University of California, Los Angeles due to its de-identified nature and informed consent was not necessary. The study was in accordance with the Strengthening the Reporting of Observational studies in Epidemiology (STROBE) guidelines.

## Results

Of 1,380,740 patients included for analysis, 1,338,417 (96.9%) survived to discharge. Compared to survivors, those who died had a greater prevalence of penetrating injuries (18.0% vs 9.44%, SMD = 0.25). As shown in Table 1, patients who died were older had higher ISS scores and more injuries. While respiratory rate was similar across groups, GCS and systolic blood pressure were lower than those who died (Table 1). Furthermore, patients who died were more commonly male sex and were more frequently insured by Medicare. They also had significantly higher rates of congestive heart failure and end stage renal disease. Patients who died were more likely managed at ACS and state designation Level I trauma centers.

**Table 1:** Demographic comparison of those who died and those who survived.

	Died (n = 42,323)	Survived (n = 1,338,417)	*p-value*	SMD
Age (years) (IQR)	61 (35–78)	51 (27–70)	<0.001	0.32
WHO Age Category (years)			<0.001	0.34
≤4	1.94	0.72		
5–14	4.61	1.39		
15–24	13.0	11.0		
25–34	13.0	10.9		
35–54	21.7	17.5		
55–74	26.1	27.9		
≥75	19.6	30.6		
Injury severity score (IQR)	25 (14–30)	6 (4–10)	<0.001	1.3
Median number of injuries (IQR)	5 (2–8)	2 (1–4)	<0.001	0.76
Glasgow Coma Scale (IQR)	4 (3–15)	15 (15–15)	<0.001	-1.66
Respiratory rate (IQR)	18 (16–22)	18 (16–20)	<0.001	0.075
Systolic blood pressure (IQR)	131 (104–156)	136 (121–153)	<0.001	-0.21
TRISS survival probability	43.6 (11.1–82.5)	98.0 (91.7–99.6)	<0.001	-1.8
Female sex	28.7	37.3	<0.001	-0.18
Insurance type			<0.001	0.30
Private	26.2	35.8		
Medicare	36.9	28.2		
Medicaid	12.2	16.7		
Self-pay	15.9	10.5		
Other/unknown	8.86	8.77		
Ethnicity			<0.001	0.08
White	66.3	67.5		
Black	14.8	14.0		
Hispanic	9.94	11.4		
Asian/Pacific islander	2.59	2.14		
Other/unknown	6.38	4.98		
Penetrating mechanism	18.0	9.44	<0.001	0.25
Mechanism stratified			<0.001	0.50
Gunshot wound	16.2	4.41		
Stabbing injury	1.79	4.97		
Blunt injury	3.6	9.1		
Fall	42.3	46.2		
Motor vehicle collision	18.4	19.7		
Motorcycle collision	7.17	8.00		
Motor vehicle vs pedestrian	10.0	6.48		
Other	0.54	1.11		
Positive blood alcohol level	12.2	11.5	<0.001	0.02
Positive illicit drug screen	9.60	10.2	<0.001	0.02
Positive prescription drug screen	5.00	5.47	<0.001	0.02
History of stroke	3.91	2.49	<0.001	0.08
Smoking history	9.34	19.6	<0.001	0.30
Chronic obstructive pulmonary disorder	8.82	6.34	<0.001	0.09
Congestive heart failure	7.23	3.21	<0.001	0.18
History of myocardial infarction	1.65	0.93	<0.001	0.06
Hypertension	33.2	30.7	<0.001	0.05
Peripheral vascular disease	1.17	0.58	<0.001	0.06
End stage renal disease	3.63	1.51	<0.001	0.13
Liver cirrhosis	2.68	0.86	<0.001	0.14
Diabetes	15.9	12.7	<0.001	0.09
Bleeding history	7.84	4.46	<0.001	0.14
Disseminated cancer	1.62	0.55	<0.001	0.10
Alcohol use disorder	6.78	6.28	<0.001	0.02
Psychiatric disorder	7.91	10.0	<0.001	0.07
History of drug use	3.72	5.78	<0.001	0.09
ADD / ADHD	0.39	1.17	<0.001	0.09
Dementia	6.07	4.10	<0.001	0.09
Advanced directive limiting care	8.65	2.15	<0.001	0.29
Dependent functional status	7.29	5.33	<0.001	0.08
ACS trauma level designation			<0.001	0.21
I	21.0	16.9		
II	11.0	10.4		
III	0.80	1.86		
Missing/not applicable	67.2	70.8		
State trauma level designation			<0.001	0.18
I	52.2	45.5		
II	28.6	28.9		
III	4.52	7.89		
IV	0.20	0.34		
Missing/not applicable	14.4	17.3		
Hospital teaching status			<0.001	0.12
University	49.4	43.8		
Non-teaching	13.8	16.7		
Community	36.7	39.3		
Missing/not applicable	0.05	0.16		
Non-profit hospital	6.20	6.15	0.63	0.002

Reported as proportions unless otherwise noted. p-values and standard mean difference (SMD) between multiple groups demonstrate significance across group. ADD/ADHD: attention deficit disorder / attention-deficit/hyperactivity disorder, WHO: World Health Organization, ACS: American College of Surgeons.

**Fig 1:**
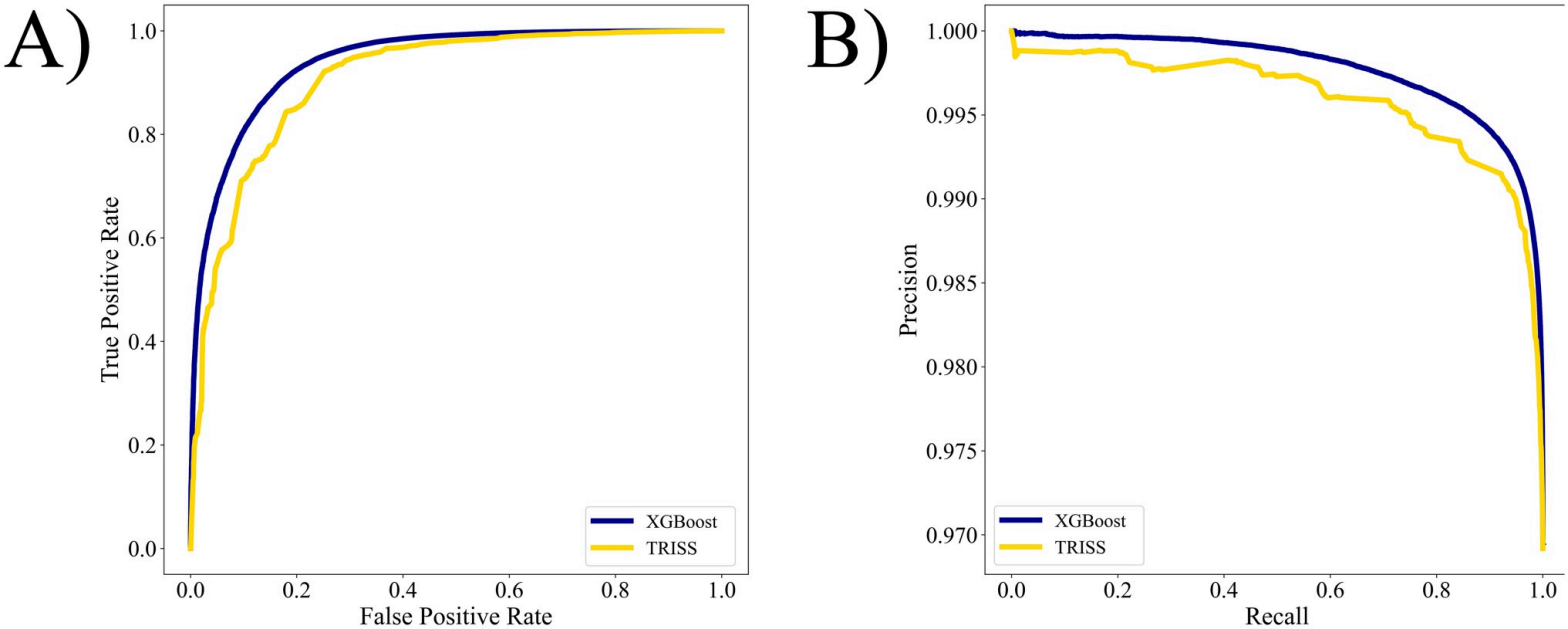
A) Area under the curve (AUC) and B) precision-recall curves comparing XGBoost and TRISS.

**Fig 2:**
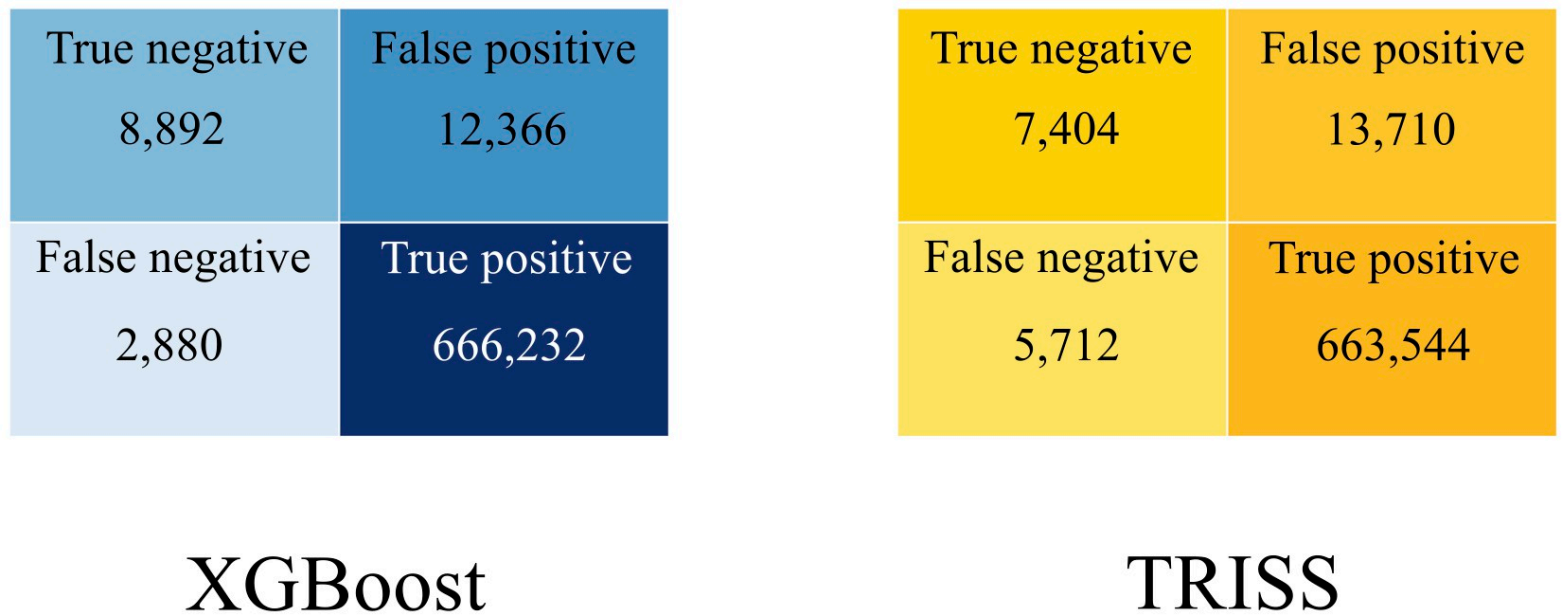
Confusion matrices of XGBoost and TRISS models demonstrating results from testing data.

**Fig 3:**
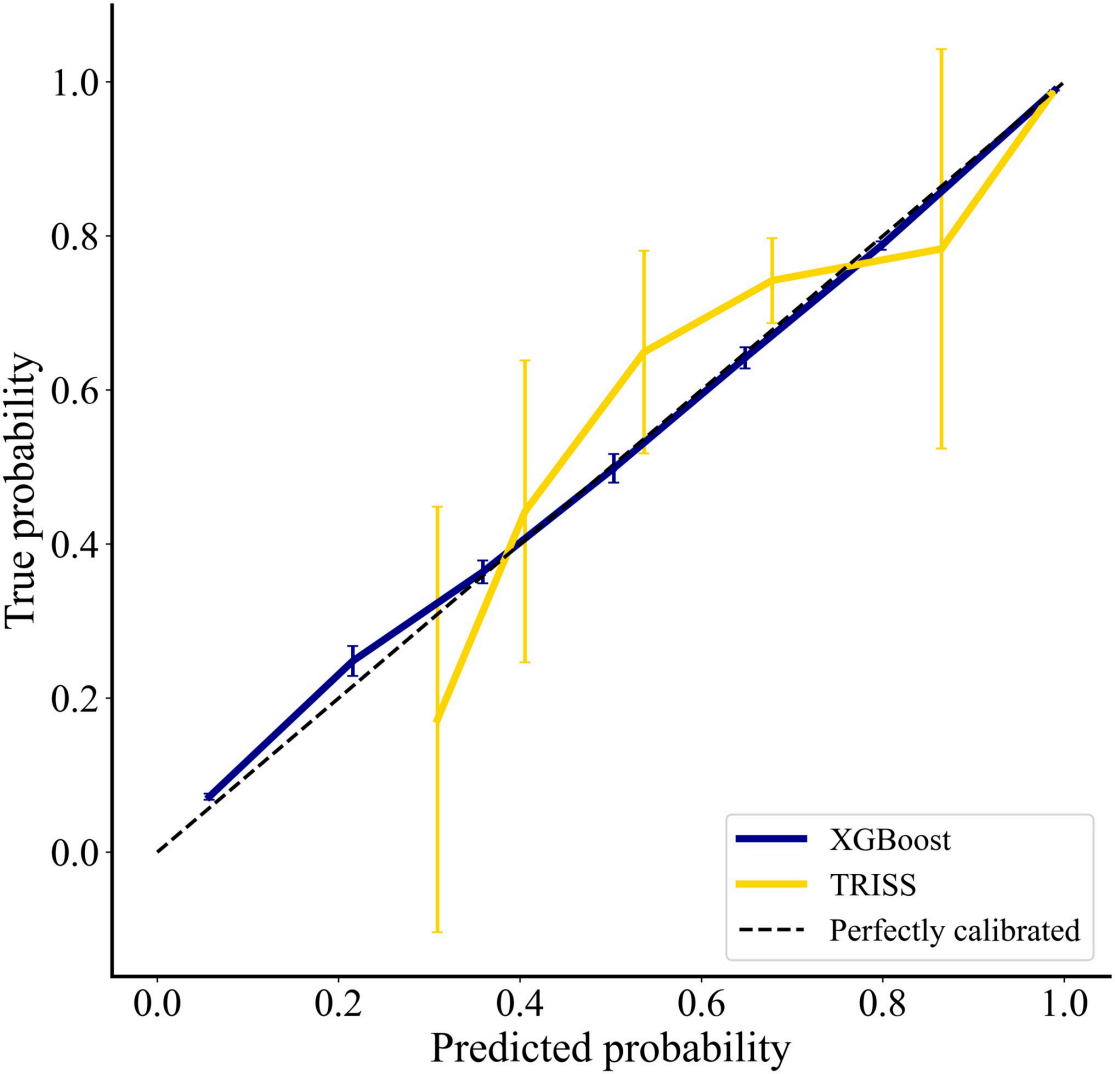
Calibration curves comparing XGBoost with TRISS.

As shown in Fig 1, the base XGBoost model demonstrated a greater ROC than TRISS (0.950 (95% CI: 0.949–0.950) vs 0.907 (95% CI: 0.907–0.907)). Additionally, greater classification accuracy, defined by improved precision and recall, was achieved by XGBoost. Compared to TRISS, the base XGBoost model correctly classified 20.1% more patients as observed in the confusion matrices (Fig 2). Superior discriminatory and classification performance for the XGBoost model persisted in all studied sub-populations (S3 Table). This model exhibited high calibration across all risk levels as demonstrated in Fig 3 (R^2^ = 0.998 vs 0.816). Notably, the large confidence intervals around the TRISS calibration curve allude to the instability of the model in predicting survival after trauma. The complete model, which consisted of injury variables, vitals, and patient demographics, exhibited a ROC of 0.960 (95% CI 0.960–0.960). Furthermore, it had exemplary calibration and precision-recall (S2 & S3 Figs).

**Fig 4:**
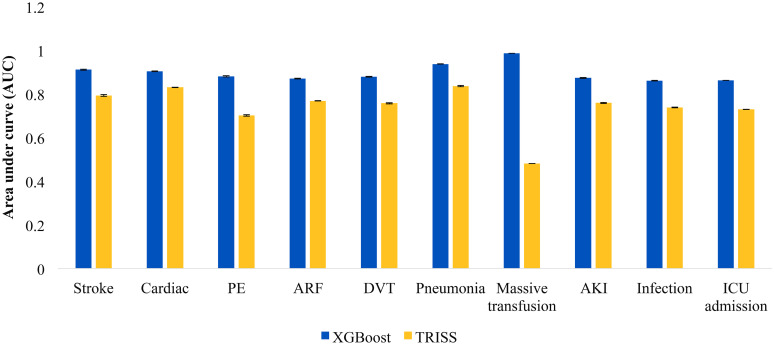
Area under the curve (AUC) of secondary outcomes of interest with corresponding 95% confidence intervals. PE: pulmonary embolism, ARF: acute respiratory failure, DVT: deep vein thrombosis, AKI: acute kidney injury, ICU: intensive care unit.

Unadjusted incidence of secondary outcomes is shown in S4 Table. On adjusted analysis (Fig 4), the base XGBoost model consistently demonstrated excellent discrimination, precision and recall compared to TRISS across all secondary outcomes (S5 Table). In particular, the model performed particularly well in the prediction of massive transfusion with a ROC of 0.986 (95% CI: 0.986–0.986). Importantly, the balanced accuracy of both TRISS and ML models were poor in most in-hospital complications. The XGBoost model did; however, have an acceptable balanced accuracy in regards to ICU admission and massive blood transfusion.

**Fig 5:**
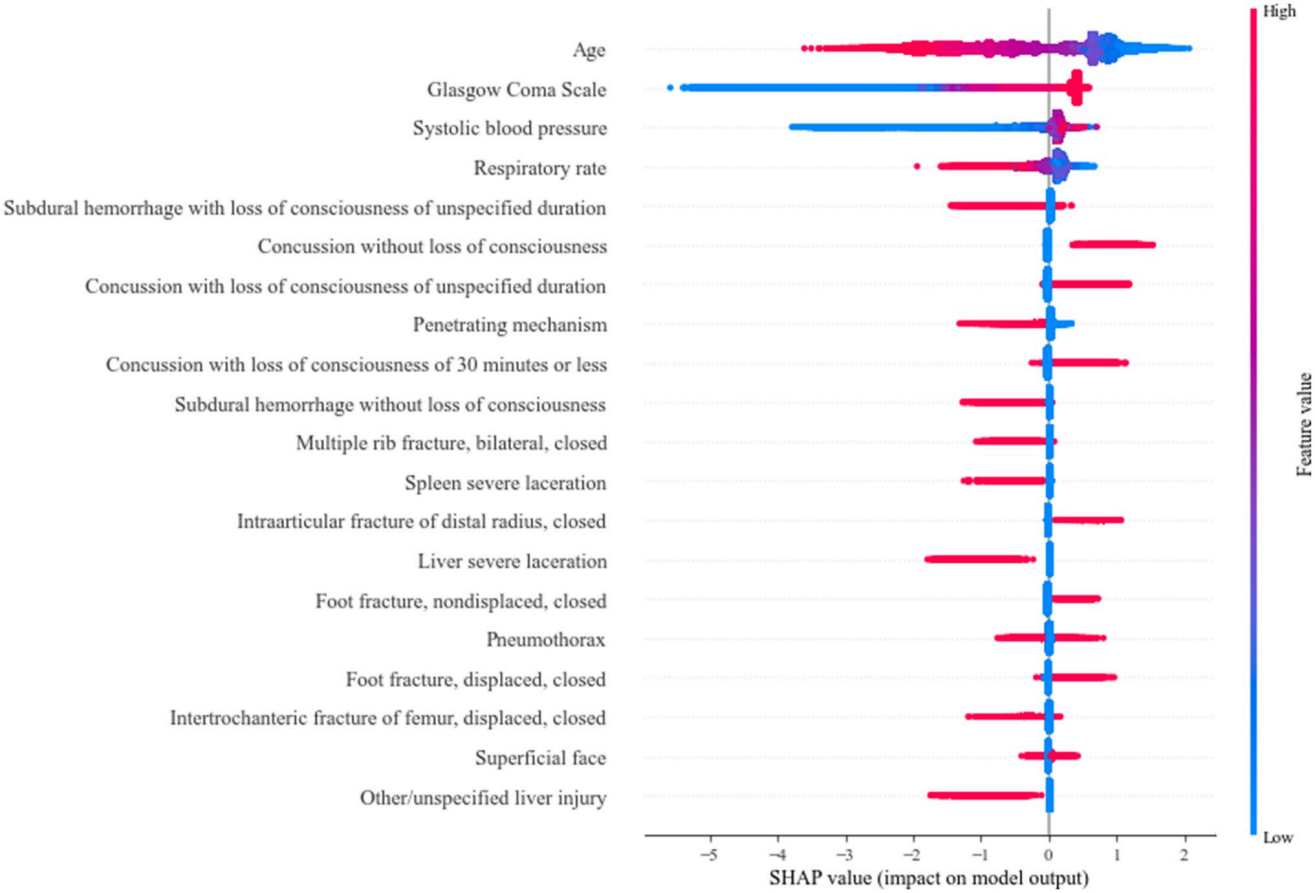
SHAP plot demonstrating the 20 most important features in the XGBoost model. Features ranked by descending importance. Red points designate higher values for that feature while blue points denote lower values.

The base XGBoost model was interpreted using SHapley summary plots, which rank the predictors of survival by their relative importance. As shown in Fig 5, red dots correspond to higher variable values, while blue dots indicate lower values. Age was the most important predictor, with younger age corresponding with improved survival. Lower GCS and SBP portended reduced survival while lower values of RR was associated with improved survival. Among the injury variables studied, head injuries were deemed of high importance, comprising 40% of the top twenty most salient features. While subdural hemorrhage was associated with mortality, concussion-related injuries were associated with survival.

Separate sensitivity analyses were performed to include those with any missing physiologic variables (14.3% of patients, n = 1,611,063). To account for missing values, imputation was used with continuous variables imputed as medians and categorical variables as the mode. As shown in S6 Table, all XGBoost models were re-analyzed and the results remained similar. Additional analyses were performed using a 60:40 training:validation split, and World Health Organization (WHO) age as a categorical value and the observed results were similar (S7 Table). In the WHO age ≥75 years subset, the performance of ML models was persistently improved compared to TRISS but was slightly diminished compared to the base model examining all ages.

## Discussion

With potential applications in benchmarking and quality improvement, mortality prediction has been of great interest in trauma. Machine learning-based models, which utilize robust mathematical methodologies and account for nonlinear relationships among covariates may provide an opportunity for improvement towards this goal. The present study used previously validated ICD-10-CM injury variables in conjunction with patient demographics and vitals to predict survival with a machine learning algorithm. Compared to the TRISS, XGBoost demonstrated significantly improved classification and calibration. Its performance was maintained across other in-hospital outcomes assessed but balanced accuracy was relatively poor. In addition, the complete XGBoost model had high performance, validating its possible utility as a mortality prediction model. Finally, we observed several patient demographics and injury features that were associated with survival. These findings warrant further discussion.

In agreement with our prior work, ML-based models were shown to have improved performance compared to preexisting injury tools [13]. These findings were anticipated given the XGBoost model’s greater ROC and better calibration following injury variable-only adjustment compared to ISS and TMPM. Greater performance is likely explained by the extensive number of features used and the decision architecture’s ability to account for multicollinearity as well as non-linear relationships. Its strengths persisted across all studied sub-populations and was augmented further following additional patient characteristics. Of note, we observed slightly diminished performance when assessing older patients (≥50 and ≥75 years) compared to the model including all ages. This may be, in part, due to diminished preinjury functional status that is not accounted in the base model [24]. Nevertheless, the present study, to our knowledge, provides the highest performance model for mortality classification to date.

In regards to secondary outcomes, the XGBoost models demonstrated overall greater performance compared to TRISS. However, it is important to consider that the balanced accuracy of ML and TRISS models were relatively poor. These findings likely relate to the skewed rates of secondary outcomes reported in the NTDB. In addition, the TRISS was created for survival prediction and has not been validated for our studied secondary outcomes. We recognize our application of TRISS was not its intended use. To date, there are no validated prediction scores present that encompass all our studied in-hospital complications. Given similar variables between both models, we sought to explore its performance to provide a comparison basis for the XGBoost models. Nevertheless, our model highlights potential applications of ML approaches beyond mortality prediction.

We observed several patient and injury characteristics to be associated with survival. Younger age, higher GCS scores and greater SBP were expectedly associated with higher likelihood of survival. Furthermore, SHapley interpretation revealed that subdural hemorrhage was associated with lower rates of survival while concussion-related injuries, including those without loss of consciousness, ≤30 minutes, or of unspecified duration, appeared to be protective. With machine learning methods and the complex interplay of injury interactions, it may be difficult to ascertain reasons for this finding. However, it is possible that relative to intracranial bleeding and other more severe head injuries, mild concussions may exhibit a protective effect in the model. Notably, our outcome evaluated in-hospital mortality and does not reflect the long-term sequelae of concussions that have been well-documented elsewhere [25–28]. Nonetheless, our findings add to the growing body of literature regarding autonomous variable selection employed by machine learning approaches that may reduce external bias and enhance generalizability.

The family of models presented herein may have several practical and important applications. First, it could be implemented into the electronic medical record and provide an updated estimate of survival over time. As the relevant injury ICD codes for the patient and as well as vitals are entered in the electronic system, the model would generate a predicted rate of mortality and other complications. While the present study evaluated the highest values within the first 24 hours of admission, an ideal model would be able to capture multiple points temporally and provide accurate estimates at any interval. With nearly perfect model calibration, our model could be applied as a risk-stratification tool that could guide resource allocation and shared decision-making. Finally, our model may have uses in hospital benchmarking. With appropriate adjustment for injury, risk adjusted outcomes could be used by initiatives such as the ACS Trauma Quality Improvement Program (TQIP) [29, 30].

Our study has several important limitations including those inherent to its retrospective nature. The NTDB is a convenience sample and is predicated on voluntary submission by trauma programs. Variable collection likely differs among institutions which may cause a large number of missing values that sensitivity analysis with simple imputation may inadequately address. Additionally, results may not be entirely generalizable to non-participating centers particularly those not in the United States. As the number of hospitals is unable to be ascertained, we were also unable to perform analysis that accounted for patient clustering within each hospital. Despite greater granularity of ICD-10 coding compared to ICD-9, 22.8% of injury variables used contained “unspecified” information. They were included in our analysis to provide the most inclusive analysis of all existing injury variables. Furthermore, injury codes in NTDB are compiled at the end of hospitalization which may limit its utility as a real-time prediction score due to reliance on accurate coding and retrospective scoring. Future studies are needed to prospectively validate these findings.

In summary, machine learning-based approaches outperform the TRISS in survival prediction following trauma-related admissions. The addition of patient comorbidities to our model resulted in exceptional discriminatory performance which persisted across risk strata. With excellent performance in prediction of several in-hospital outcomes, our findings further demonstrate the value of machine learning algorithms in trauma.

## Supporting information

S1 Table NTDB-provided demographic and comorbidities used in complete XGBoost model. Patients with unlisted/unspecified insurance type, ethnicity, or mechanism were denoted as “other/unknown.” ADD/ADHD: attention deficit disorder / attention-deficit/hyperactivity disorder, ACS: American College of Surgeons *Positive drug screens in the NTDB contain numerous, variable permutations (not tested, negative, not applicable, not recorded, trace levels, and beyond legal limit). To simplify analysis, these variables were simplified to binary factors with “beyond legal limit” denoted as positive and all other values deemed negative.
(DOCX)

S2 Table Hyperparameters used in the XGBoost models. (DOCX)

S3 Table Performance metrics of XGBoost and TRISS models with sub-populations included. All metrics shown with corresponding 95% confidence intervals. Patient counts reported for those in testing data. AUC: area under curve.
(DOCX)

S4 Table Unadjusted rates of secondary outcomes. ICU: intensive care unit.
(DOCX)

S5 Table Performance metrics of XGBoost and TRISS for studied secondary outcomes with corresponding 95% confidence intervals. Patient counts reported for those in testing data. AKI: acute kidney injury, PE: pulmonary embolism, ARF: acute respiratory failure, DVT: deep vein thrombosis, ICU: intensive care unit.
(DOCX)

S6 Table Sensitivity analyses of XGBoost models for all outcomes following imputation with corresponding 95% confidence intervals. Patient counts reported for those in testing data. AKI: acute kidney injury, PE: pulmonary embolism, ARF: acute respiratory failure, DVT: deep vein thrombosis, ICU: intensive care unit.
(DOCX)

S7 Table Additional sensitivity analyses of XGBoost models for parameters shown. Patient counts reported for those in testing data. WHO: World Health Organization.
(DOCX)

S1 Fig Schematic demonstrating variables used in each model. ISS: Injury Severity Score, ML: machine learning, RR: respiratory rate, SBP: systolic blood pressure, GCS: Glasgow Coma Scale.
(DOCX)

S2 Fig Area under curve and precision-recall of full XGBoost model. AUC: 0.960.
(DOCX)

S3 Fig Calibration curve of full XGBoost model. R^2^: 0.998.
(DOCX)

## References

[1] Roth GA, Abate D, Abate KH, Abay SM, Abbafati C, Abbasi N, et al. Global, regional, and national age-sex-specific mortality for 282 causes of death in 195 countries and territories, 1980–2017: a systematic analysis for the Global Burden of Disease Study 2017. *Lancet*. 2018;392(10159):1736–88. doi: 10.1016/S0140-6736(18)32203-7 30496103 PMC6227606

[2] Sewalt CA, Venema E, Wiegers EJA, Lecky FE, Schuit SCE, den Hartog D, et al. Trauma models to identify major trauma and mortality in the prehospital setting. *Br J Surg*. 2020;107:373–80. doi: 10.1002/bjs.11304 31503341 PMC7079101

[3] Cook A, Weddle J, Baker S, Hosmer D, Glance L, Friedman L, et al. A comparison of the Injury Severity Score and the Trauma Mortality Prediction Model. *J Trauma Acute Care Surg*. 2014;76:47–53. doi: 10.1097/TA.0b013e3182ab0d5d 24368356

[4] Lavoie A, Moore L, LeSage N, Liberman M, Sampalis JS. The New Injury Severity Score: A More Accurate Predictor of In-Hospital Mortality than the Injury Severity Score. *J Trauma Inj Infect Crit Care*. 2004;56:1312–20. 10.1097/01.ta.0000075342.36072.ef 15211142

[5] Linn S. The injury severity score-Importance and uses. *Ann Epidemiol*. 1995;5(6):440–446. doi: 10.1016/1047-2797(95)00059-3 8680606

[6] Gabbe BJ, Cameron PA, Wolfe R. TRISS: Does It Get Better than This? *Acad Emerg Med*. 2004;11(2):181–186. 14759963

[7] Boyd CR, Tolson MA, Copes WS. Evaluating trauma care: The TRISS method. *J Trauma—Inj Infect Crit Care*. 1987;27(4):370–378. 3106646

[8] Osler TM, Glance LG, Cook A, Buzas JS, Hosmer DW. A trauma mortality prediction model based on the ICD-10-CM lexicon: TMPM-ICD10. *J Trauma Acute Care Surg*. 2019;86(5):891–895. doi: 10.1097/TA.0000000000002194 30633101

[9] Glance LG, Osler TM, Mukamel DB, Meredith W, Wagner J, Dick AW. TMPM–ICD9. *Ann Surg*. 2009;249(6):1032–1039. 19474696 10.1097/SLA.0b013e3181a38f28

[10] Hadaya J, Verma A, Sanaiha Y, Ramezani R, Qadir N, Benharash P. Machine learning-based modeling of acute respiratory failure following emergency general surgery operations. Pasin L, ed. *PLoS One*. 2022;17(4):e0267733. doi: 10.1371/journal.pone.0267733 35482751 PMC9049563

[11] Verma A, Sanaiha Y, Hadaya J, Maltagliati AJ, Tran Z, Ramezani R, et al. Parsimonious machine learning models to predict resource use in cardiac surgery across a statewide collaborative. *JTCVS Open*. Published online April 20, 2022. doi: 10.1016/j.xjon.2022.04.017 36172420 PMC9510828

[12] Elfanagely O, Toyoda Y, Othman S, Mellia JA, Basta M, Liu T, et al. Machine learning and surgical outcomes prediction: a systematic review. *J*. *Surg*. *Res*. 2021;264:346–61. doi: 10.1016/j.jss.2021.02.045 33848833

[13] Tran Z, Zhang W, Verma A, Cook A, Kim D, Burruss S, et al. The derivation of an International Classification of Diseases, Tenth Revision–based trauma-related mortality model using machine learning. *J Trauma Acute Care Surg*. 2022; 92(3):561–6. doi: 10.1097/TA.0000000000003416 34554135

[14] Brooks SE, Mukherjee K, Gunter OL, Guillamondegui OD, Jenkins JM, Miller RS, et al. Do Models Incorporating Comorbidities Outperform Those Incorporating Vital Signs and Injury Pattern for Predicting Mortality in Geriatric Trauma? *J Am Coll Surg*. 2014;219(5):1020–1027. doi: 10.1016/j.jamcollsurg.2014.08.001 25260686

[15] Mukherjee K, Rimer M, McConnell MD, Miller RS, Morrow SE. Physiologically focused triage criteria improve utilization of pediatric surgeon-directed trauma teams and reduce costs. *J Pediatr Surg*. 2010;45(6):1315–1323. doi: 10.1016/j.jpedsurg.2010.02.108 20620338

[16] Madrigal J, Mukdad L, Han AY, Tran Z, Benharash P, St. John MA, et al. Impact of Hospital Volume on Outcomes Following Head and Neck Cancer Surgery and Flap Reconstruction. *Laryngoscope*. 2022;132(7):1381–1387. doi: 10.1002/lary.29903 34636433

[17] Chen T, Guestrin C. XGBoost: A scalable tree boosting system. In: *Proceedings of the ACM SIGKDD International Conference on Knowledge Discovery and Data Mining*. Vol 13-17-August-2016. Association for Computing Machinery; 2016:785–794.

[18] *Python API Reference—xgboost 0*.*82 documentation*. Accessed October 7, 2022. https://xgboost.readthedocs.io/en/release_0.82/python/python_api.html.

[19] Boyd K, Eng KH, Page CD. Area under the precision-recall curve: Point estimates and confidence intervals. In: *Lecture Notes in Computer Science (Including Subseries Lecture Notes in Artificial Intelligence and Lecture Notes in Bioinformatics)*. Vol 8190 LNAI. Springer, Berlin, Heidelberg; 2013:451–466.

[20] Rufibach K. Use of Brier score to assess binary predictions. *J Clin Epidemiol*. 2010;63(8):938–939. doi: 10.1016/j.jclinepi.2009.11.009 20189763

[21] Lundberg SM, Allen PG, Lee SI. A Unified Approach to Interpreting Model Predictions. Accessed November 27, 2021. https://github.com/slundberg/shap.

[22] Mckinney W. *Pandas*: *A Foundational Python Library for Data Analysis and Statistics*. Accessed June 14, 2021. http://pandas.sf.net.

[23] Pedregosa F, Michel V, Grisel O, Blondel M, Prettenhofer P, Weiss R, et al. *Scikit-Learn*: *Machine Learning in Python Gaël Varoquaux Bertrand Thirion Vincent Dubourg Alexandre Passos PEDREGOSA*, *VAROQUAUX*, *GRAMFORT ET AL*. *Matthieu Perrot*. Vol 12.; 2011. Accessed May 17, 2021. http://scikit-learn.sourceforge.net.

[24] Maxwell CA, Mion LC, Mukherjee K, Dietrich MS, Minnick A, May A, et al. Preinjury physical frailty and cognitive impairment among geriatric trauma patients determine postinjury functional recovery and survival. *J Trauma Acute Care Surg*. 2016;80(2):195–203. doi: 10.1097/TA.0000000000000929 26595712

[25] Wilson L, Stewart W, Dams-O’Connor K, Diaz-Arrastia R, Horton L, Menon DK, et al. The chronic and evolving neurological consequences of traumatic brain injury. *Lancet Neurol*. 2017;16(10):813–825. doi: 10.1016/S1474-4422(17)30279-X 28920887 PMC9336016

[26] Badri S, Chen J, Barber J, Temkin NR, Dikmen SS, Chesnut RM, et al. Mortality and long-term functional outcome associated with intracranial pressure after traumatic brain injury. *Intensive Care Med*. 2012;38(11):1800–1809. doi: 10.1007/s00134-012-2655-4 23011528

[27] Raj R, Luostarinen T, Pursiainen E, Posti JP, Takala RSK, Bendel S, et al. Machine learning-based dynamic mortality prediction after traumatic brain injury. *Sci Rep*. 2019;9(1):1–13. 31776366 10.1038/s41598-019-53889-6 PMC6881446

[28] Rau CS, Kuo PJ, Chien PC, Huang CY, Hsieh HY, Hsieh CH. Mortality prediction in patients with isolated moderate and severe traumatic brain injury using machine learning models. Kou YR, ed. *PLoS One*. 2018;13(11):e0207192. doi: 10.1371/journal.pone.0207192 30412613 PMC6226171

[29] Newgard CD, Fildes JJ, Wu L, Hemmila MR, Burd RS, Neal M, et al. Methodology and Analytic Rationale for the American College of Surgeons Trauma Quality Improvement Program. *J Am Coll Surg*. 2013;216(1):147–157. doi: 10.1016/j.jamcollsurg.2012.08.017 23062519

[30] Hornor MA, Hoeft C, Nathens AB. Quality Benchmarking in Trauma: from the NTDB to TQIP. *Curr Trauma Reports*. 2018;4(2):160–169.

