# Supplementary material for: ICD-10 based machine learning models outperform the Trauma and Injury Severity Score (TRISS) in survival prediction
Source: PLoS One. 2022 Oct 27;17(10):e0276624. doi: 10.1371/journal.pone.0276624 (PMC9612528; doi:10.1371/journal.pone.0276624)
Supplement: S1 Table — Patients with unlisted/unspecified insurance type, ethnicity, or mechanism were denoted as “other/unknown.” ADD/ADHD: attention deficit disorder / attention-deficit/hyperactivity disorder, ACS: American College of Surgeons *Positive drug screens in the NTDB contain numerous, variable permutations (not tested, negative, not applicable, not recorded, trace levels, and beyond legal limit). To simplify analysis, these variables were simplified to binary factors with “beyond legal limit” denoted as positive and all other values deemed negative. (DOCX) [file pone.0276624.s001.docx]

| **NTDB-provided demographic and comorbidities** |
| --- |
| Age |
| Female sex |
| Insurance type |
| Private |
| Medicare |
| Medicaid |
| Self-pay |
| Other/unknown |
| Ethnicity |
| White |
| Black |
| Hispanic |
| Asian/Pacific islander |
| Other/unknown |
| Mechanism |
| Gunshot wound |
| Blunt injury |
| Fall |
| Motor vehicle collision |
| Motorcycle collision |
| Motor vehicle vs pedestrian |
| Stabbing injury |
| Other/unknown |
| *Positive blood alcohol level |
| *Positive illicit drug screen |
| *Positive prescription drug screen |
| History of stroke |
| Smoking history |
| Chronic obstructive pulmonary disorder |
| Congestive heart failure |
| History of myocardial infarction |
| Hypertension |
| Peripheral vascular disease |
| End stage renal disease |
| Liver cirrhosis |
| Diabetes |
| Bleeding history |
| Disseminated cancer |
| Alcohol use disorder |
| Psychiatric disorder |
| History of drug use |
| ADD / ADHD |
| Dementia |
| Advanced directive limiting care |
| Dependent functional status |
| Chemotherapy history |
| Steroid use |
| Angina pectoris |
| Congenital abnormality |
| Prematurity |
| ACS trauma level designation |
| I |
| II |
| III |
| State trauma level designation |
| I |
| II |
| III |
| IV |
| Hospital teaching status |
| University |
| Non-teaching |
| Community |
| Non-profit hospital |

S1 Table. NTDB-provided demographic and comorbidities used in complete XGBoost model. Patients with unlisted/unspecified insurance type, ethnicity, or mechanism were denoted as “other/unknown.” ADD/ADHD: attention deficit disorder / attention-deficit/hyperactivity disorder, ACS: American College of Surgeons *Positive drug screens in the NTDB contain numerous, variable permutations (not tested, negative, not applicable, not recorded, trace levels, and beyond legal limit). To simplify analysis, these variables were simplified to binary factors with “beyond legal limit” denoted as positive and all other values deemed negative.
