# Supplementary material for: ICD-10 based machine learning models outperform the Trauma and Injury Severity Score (TRISS) in survival prediction
Source: PLoS One. 2022 Oct 27;17(10):e0276624. doi: 10.1371/journal.pone.0276624 (PMC9612528; doi:10.1371/journal.pone.0276624)
Supplement: S2 Table — (DOCX) [file pone.0276624.s002.docx]

| **Hyperparameter** | **Value** |
| --- | --- |
| n_estimators | 100 |
| max_depth | 3 |
| subsample | 1 |
| colsample_bytree | 1 |
| min_child_weight | 1 |
| learning_rate | 0.1 |
| gamma | 0 |
| max_delta_step | 0 |
| reg_alpha | 0 |
| reg_lambda | 1 |
| scale_pos_weight | 1 |

S2 Table. Hyperparameters used in the XGBoost models.
