# Supplementary material for: ICD-10 based machine learning models outperform the Trauma and Injury Severity Score (TRISS) in survival prediction
Source: PLoS One. 2022 Oct 27;17(10):e0276624. doi: 10.1371/journal.pone.0276624 (PMC9612528; doi:10.1371/journal.pone.0276624)
Supplement: S3 Table — All metrics shown with corresponding 95% confidence intervals. Patient counts reported for those in testing data. AUC: area under curve. (DOCX) [file pone.0276624.s003.docx]

| **Metric** | **Base Model**  **(n = 690,370)** |  | **Head**  **(n = 249,477)** |  | **Non-Head**  **(n = 440,892)** |  | **Blunt**  **(n = 623,372)** |  | **Penetrating**  **(n = 66,998)** |  | **Old**  **(n = 356,888)** |  | **Young**  **(n = 333,482)** |
| --- | --- | --- | --- | --- | --- | --- | --- | --- | --- | --- | --- | --- | --- |
|  |  |  |  |  |  |  |  |  |  |  |  |  |  |
| **XGBoost** |  |  |  |  |  |  |  |  |  |  |  |  |  |
| AUC | 0.950 (0.949-0.950) |  | 0.959 (0.959-0.959) |  | 0.930 (0.929-0.931) |  | 0.943 (0.943-0.943) |  | 0.989 (0.989-0.989) |  | 0.915 (0.914-0.916) |  | 0.983 (0.983-0.983) |
| Recall | 0.997 (0.997-0.997) |  | 0.992 (0.992-0.992) |  | 1.000 (0.999-1.000) |  | 0.997 (0.997-0.997) |  | 0.996 (0.996-0.996) |  | 0.996 (0.996-0.996) |  | 0.998 (0.998-0.998) |
| Precision | 0.983 (0.983-0.983) |  | 0.973 (0.973-0.973) |  | 0.999 (0.999-0.999) |  | 0.983 (0.983-0.983) |  | 0.988 (0.988-0.988) |  | 0.976 (0.976-0.976) |  | 0.991 (0.991-0.991) |
| Specificity | 0.421 (0.420-0.422) |  | 0.503 (0.499-0.507) |  | 0.272 (0.269-0.275) |  | 0.361 (0.359-0.362) |  | 0.717 (0.714-0.721) |  | 0.339 (0.337-0.342) |  | 0.561 (0.559-0.564) |
| Balanced Accuracy | 0.726 (0.726-0.727) |  | 0.771 (0.770-0.772) |  | 0.665 (0.664-0.666) |  | 0.699 (0.698-0.700) |  | 0.895 (0.894-0.896) |  | 0.692 (0.691-0.693) |  | 0.815 (0.813-0.817) |
| Brier Score | 0.020 (0.020-0.020) |  | 0.034 (0.034-0.034) |  | 0.011 (0.01-0.011) |  | 0.020 (0.019-0.020) |  | 0.016 (0.015-0.016) |  | 0.027 (0.027-0.027) |  | 0.011 (0.010-0.011) |
|  |  |  |  |  |  |  |  |  |  |  |  |  |  |
| **TRISS** |  |  |  |  |  |  |  |  |  |  |  |  |  |
| AUC | 0.907 (0.907-0.907) |  | 0.916 (0.915-0.916) |  | 0.859 (0.858-0.86) |  | 0.895 (0.894-0.896) |  | 0.963 (0.962-0.964) |  | 0.849 (0.848-0.849) |  | 0.954 (0.954-0.954) |
| Recall | 0.992 (0.992-0.992) |  | 0.982 (0.982-0.982) |  | 0.997 (0.997-0.997) |  | 0.994 (0.994-0.994) |  | 0.981 (0.981-0.981) |  | 0.991 (0.991-0.991) |  | 0.995 (0.994-0.995) |
| Precision | 0.980 (0.980-0.980) |  | 0.965 (0.965-0.965) |  | 0.988 (0.988-0.988) |  | 0.979 (0.979-0.979) |  | 0.979 (0.979-0.979) |  | 0.097 (0.097-0.097) |  | 0.985 (0.985-0.985) |
| Specificity | 0.348 (0.347-0.349) |  | 0.414 (0.411-0.418) |  | 0.190 (0.188-0.193) |  | 0.249 (0.248-0.251) |  | 0.656 (0.652-0.660) |  | 0.322 (0.320-0.324) |  | 0.337 (0.335-0.339) |
| Balanced Accuracy | 0.670 (0.669-0.671) |  | 0.699 (0.698-0.700) |  | 0.594 (0.593-0.595) |  | 0.622 (0.621-0.623) |  | 0.819 (0.817-0.821) |  | 0.656 (0.655-0.657) |  | 0.666 (0.665-0.667) |
| Brier Score | 0.028 (0.028-0.028) |  | 0.051 (0.051-0.051) |  | 0.015 (0.015-0.015) |  | 0.027 (0.027-0.027) |  | 0.037 (0.037-0.037) |  | 0.034 (0.034-0.034) |  | 0.021 (0.020-0.021) |

S3 Table. Performance metrics of XGBoost and TRISS models with sub-populations included. All metrics shown with corresponding 95% confidence intervals. Patient counts reported for those in testing data. Patient counts reported for those in testing data. AUC: area under curve
