# Supplementary material for: ICD-10 based machine learning models outperform the Trauma and Injury Severity Score (TRISS) in survival prediction
Source: PLoS One. 2022 Oct 27;17(10):e0276624. doi: 10.1371/journal.pone.0276624 (PMC9612528; doi:10.1371/journal.pone.0276624)
Supplement: S4 Table — ICU: intensive care unit. (DOCX) [file pone.0276624.s004.docx]

| **Outcome** | **n** | **Rate (%)** |
| --- | --- | --- |
| Stroke | 3,046 | 0.22 |
| Cardiac | 10,247 | 0.74 |
| Pulmonary embolism | 4,026 | 0.29 |
| Acute respiratory failure | 16,081 | 1.16 |
| Deep vein thrombosis | 8,061 | 0.58 |
| Pneumonia | 7,154 | 0.52 |
| Massive Transfusion | 171,717 | 12.4 |
| Acute kidney injury | 6,937 | 0.50 |
| Infection | 11,500 | 0.83 |
| ICU admission | 382,491 | 27.7 |

S4 Table. Unadjusted rates of secondary outcomes. ICU: intensive care unit
