# Supplementary material for: ICD-10 based machine learning models outperform the Trauma and Injury Severity Score (TRISS) in survival prediction
Source: PLoS One. 2022 Oct 27;17(10):e0276624. doi: 10.1371/journal.pone.0276624 (PMC9612528; doi:10.1371/journal.pone.0276624)
Supplement: S5 Table — Patient counts reported for those in testing data. AKI: acute kidney injury, PE: pulmonary embolism, ARF: acute respiratory failure, DVT: deep vein thrombosis, ICU: intensive care unit. (DOCX) [file pone.0276624.s005.docx]

| **Metric** | **Stroke**  **(n = 1,523)** | **Cardiac**  **(n = 5,123)** | **PE**  **(n = 2,013)** | **ARF**  **(n = 8,040)** | **DVT**  **(n = 4,030)** |
| --- | --- | --- | --- | --- | --- |
|  |  |  |  |  |  |
| **XGBoost** |  |  |  |  |  |
| AUC | 0.911 (0.909-0.913) | 0.904 (0.903-0.905) | 0.881 (0.879-0.883) | 0.870 (0.869-0.871) | 0.878 (0.877-0.879) |
| Recall | 0.037 (0.033-0.040) | 0.050 (0.048-0.052) | 0.023 (0.021-0.025) | 0.024 (0.023-0.025) | 0.004 (0.002-0.005) |
| Precision | 0.991 (0.985-0.997) | 0.950 (0.942-0.958) | 0.990 (0.982-0.998) | 0.910 (0.895-0.925) | 0.013 (0.005-0.021) |
| Specificity | 1.000 (1.000-1.000) | 1.000 (1.000-1.000) | 1.000 (1.000-1.000) | 1.000 (1.000-1.000) | 1.000 (1.000-1.000) |
| Balanced Accuracy | 0.518 (0.516-0.520) | 0.524 (0.523-0.525) | 0.512 (0.511-0.513) | 0.512 (0.511-0.513) | 0.516 (0.515-0.516) |
| Brier Score | 0.002 (0.002-0.002) | 0.007 (0.006-0.008) | 0.003 (0.003-0.003) | 0.011 (0.011-0.011) | 0.006 (0.006-0.006) |
|  |  |  |  |  |  |
| **TRISS** |  |  |  |  |  |
| AUC | 0.793 (0.791-0.796) | 0.830 (0.829-0.831) | 0.701 (0.699-0.703) | 0.768 (0.768-0.768) | 0.757 (0.755-0.759) |
| Recall | 0.000 (0.000-0.000) | 0.000 (0.000-0.000) | 0.000 (0.000-0.000) | 0.000 (0.000-0.000) | 0.000 (0.000-0.000) |
| Precision | 0.000 (0.000-0.000) | 0.000 (0.000-0.000) | 0.000 (0.000-0.000) | 0.000 (0.000-0.000) | 0.000 (0.000-0.000) |
| Specificity | 1.000 (1.000-1.000) | 1.000 (1.000-1.000) | 1.000 (1.000-1.000) | 1.000 (1.000-1.000) | 1.000 (1.000-1.000) |
| Balanced Accuracy | 0.500 (0.500-0.500) | 0.500 (0.500-0.500) | 0.500 (0.500-0.500) | 0.500 (0.500-0.500) | 0.500 (0.500-0.500) |
| Brier Score | 0.002 (0.002-0.002) | 0.007 (0.007-0.007) | 0.003 (0.003-0.003) | 0.012 (0.012-0.012) | 0.006 (0.006-0.006) |
|  |  |  |  |  |  |
|  |  |  |  |  |  |
| **Metric** | **Pneumonia**  **(n = 3,577)** | **Massive Transfusion**  **(n = 85,858)** | **AKI**  **(n= 3,468)** | **Infection**  **(n = 5,750)** | **ICU admission**  **(n = 191,245)** |
|  |  |  |  |  |  |
| **XGBoost** |  |  |  |  |  |
| AUC | 0.937 (0.936-0.938) | 0.986 (0.986-0.986) | 0.873 (0.872-0.874) | 0.861 (0.860-0.862) | 0.862 (0.862-0.862) |
| Recall | 0.052 (0.050-0.054) | 0.336 (0.330-0.342) | 0.041 (0.040-0.042) | 0.037 (0.036-0.038) | 0.551 (0.549-0.552) |
| Precision | 0.970 (0.964-0.976) | 0.859 (0.854-0.864) | 0.969 (0.963-0.975) | 0.937 (0.928-0.946) | 0.767 (0.766-0.767) |
| Specificity | 1.000 (1.000-1.000) | 0.999 (0.999-0.999) | 1.000 (1.000-1.000) | 1.000 (1.000-1.000) | 0.933 (0.933-0.934) |
| Balanced Accuracy | 0.526 (0.525-0.527) | 0.668 (0.665-0.671) | 0.521 (0.520-0.522) | 0.519 (0.519-0.519) | 0.743 (0.742-0.744) |
| Brier Score | 0.005 (0.004-0.006) | 0.005 (0.005-0.005) | 0.005 (0.004-0.006) | 0.008 (0.008-0.008) | 0.171 (0.171-0.171) |
|  |  |  |  |  |  |
| **TRISS** |  |  |  |  |  |
| AUC | 0.836 (0.834-0.838) | 0.481 (0.48-0.481) | 0.759 (0.757-0.761) | 0.738 (0.737-0.739) | 0.729 (0.729-0.729) |
| Recall | 0.000 (0.000-0.000) | 0.000 (0.000-0.000) | 0.000 (0.000-0.000) | 0.000 (0.000-0.000) | 0.207 (0.206-0.207) |
| Precision | 0.000 (0.000-0.000) | 0.000 (0.000-0.000) | 0.000 (0.000-0.000) | 0.000 (0.000-0.000) | 0.820 (0.819-0.821) |
| Specificity | 1.000 (1.000-1.000) | 1.000 (1.000-1.000) | 1.000 (1.000-1.000) | 1.000 (1.000-1.000) | 0.979 (0.979-0.979) |
| Balanced Accuracy | 0.500 (0.500-0.500) | 0.500 (0.500-0.500) | 0.500 (0.500-0.500) | 0.500 (0.500-0.500) | 0.595 (0.594-0.595) |
| Brier Score | 0.005 (0.005-0.005) | 0.124 (0.124-0.124) | 0.005 (0.005-0.005) | 0.008 (0.008-0.008) | 0.023 (0.023-0.023) |

S5 Table. Performance metrics of XGBoost and TRISS for studied secondary outcomes with corresponding 95% confidence intervals. Patient counts reported for those in testing data. AKI: acute kidney injury, PE: pulmonary embolism, ARF: acute respiratory failure, DVT: deep vein thrombosis, ICU: intensive care unit
