# Supplementary material for: ICD-10 based machine learning models outperform the Trauma and Injury Severity Score (TRISS) in survival prediction
Source: PLoS One. 2022 Oct 27;17(10):e0276624. doi: 10.1371/journal.pone.0276624 (PMC9612528; doi:10.1371/journal.pone.0276624)
Supplement: S6 Table — Patient counts reported for those in testing data. AKI: acute kidney injury, PE: pulmonary embolism, ARF: acute respiratory failure, DVT: deep vein thrombosis, ICU: intensive care unit. (DOCX) [file pone.0276624.s006.docx]

| **Metric** | **Base model**  **(n = 778,096)** |  | | **Full model**  **(n = 778,096)** | | |  | **Stroke**  **(n = 1,820)** | | |  | **Cardiac**  **(n = 6,369)** | | |  | **PE**  **(n = 2,229)** | | |  | **ARF**  **(n = 9,104)** | |
| --- | --- | --- | --- | --- | --- | --- | --- | --- | --- | --- | --- | --- | --- | --- | --- | --- | --- | --- | --- | --- | --- |
|  |  |  | |  | | |  |  | | |  |  | | |  |  | | |  |  | |
| AUC | 0.934 (0.934-0.935) |  | | 0.945 (0.945-0.945) | | |  | 0.816 (0.815-0.818) | | |  | 0.862 (0.861-0.863) | | |  | 0.786 (0.784-0.788) | | |  | 0.826 (0.825-0.827) | |
| Recall | 0.996 (0.996-0.996) |  | | 0.995 (0.995-0.995) | | |  | 0.001 (0.001-0.001) | | |  | 0.007 (0.007-0.008) | | |  | 0.000 (0.000-0.001) | | |  | 0.002 (0.002-0.003) | |
| Precision | 0.980 (0.980-0.980) |  | | 0.980 (0.980-0.981) | | |  | 0.090 (0.056-0.124) | | |  | 0.238 (0.215-0.260) | | |  | 0.049 (0.023-0.076) | | |  | 0.160 (0.143-0.176) | |
| Specificity | 0.416 (0.415-0.418) |  | | 0.437 (0.435-0.439) | | |  | 1.000 (1.000-1.000) | | |  | 1.000 (1.000-1.000) | | |  | 1.000 (1.000-1.000) | | |  | 1.000 (1.000-1.000) | |
| Balanced Accuracy | 0.706 (0.705-0.707) |  | | 0.716 (0.715-0.717) | | |  | 0.500 (0.500-0.501) | | |  | 0.504 (0.503-0.504) | | |  | 0.500 (0.500-0.500) | | |  | 0.501 (0.501-0.501) | |
| Brier Score | 0.024 (0.024-0.024) |  | | 0.024 (0.024-0.024) | | |  | 0.002 (0.002-0.002) | | |  | 0.008 (0.008-0.008) | | |  | 0.003 (0.003-0.003) | | |  | 0.011 (0.011-0.011) | |
|  |  |  | |  | | |  |  | | |  |  | | |  |  | | |  |  | |
| **Metric** | **DVT**  **(n = 4,518)** |  | **Pneumonia**  **(n = 3,982)** | |  | **Massive transfusion**  **(n = 3,860)** | | | | **AKI**  **(n = 4,110)** | | |  | **Infection**  **(n = 6,483)** | | |  | **ICU admission**  **(n = 217,588)** | | |  |
|  |  |  |  | |  |  | | |  |  | | |  |  | | |  |  | | |  |
| AUC | 0.818 (0.817-0.820) |  | 0.900 (0.899-0.902) | |  | 0.975 (0.975-0.975) | | |  | 0.801 (0.800-0.802) | | |  | 0.806 (0.805-0.808) | | |  | 0.854 (0.853-0.854) | | |  |
| Recall | 0.003 (0.002-0.003) |  | 0.005 (0.005-0.006) | |  | 0.104 (0.101-0.106) | | |  | 0.003 (0.002-0.003) | | |  | 0.005 (0.005-0.006) | | |  | 0.532 (0.531-0.533) | | |  |
| Precision | 0.156 (0.122-0.189) |  | 0.185 (0.173-0.198) | |  | 0.408 (0.399-0.418) | | |  | 0.172 (0.132-0.212) | | |  | 0.210 (0.199-0.221) | | |  | 0.750 (0.750-0.751) | | |  |
| Specificity | 1.000 (1.000-1.000) |  | 1.000 (1.000-1.000) | |  | 0.999 (0.999-0.999) | | |  | 1.000 (1.000-1.000) | | |  | 1.000 (1.000-1.000) | | |  | 0.934 (0.934-0.935) | | |  |
| Balanced Accuracy | 0.501 (0.501-0.501) |  | 0.503 (0.503-0.503) | |  | 0.551 (0.550-0.553) | | |  | 0.501 (0.501-0.502) | | |  | 0.502 (0.502-0.503) | | |  | 0.733 (0.733-0.734) | | |  |
| Brier Score | 0.006 (0.006-0.006) |  | 0.005 (0.005-0.005) | |  | 0.005 (0.005-0.005) | | |  | 0.005 (0.005-0.005) | | |  | 0.008 (0.008-0.008) | | |  | 0.174 (0.174-0.174) | | |  |

S6 Table. Sensitivity analyses of XGBoost models for all outcomes following imputation with corresponding 95% confidence intervals. Patient counts reported for those in testing data. AKI: acute kidney injury, PE: pulmonary embolism, ARF: acute respiratory failure, DVT: deep vein thrombosis, ICU: intensive care unit
