# Supplementary material for: ICD-10 based machine learning models outperform the Trauma and Injury Severity Score (TRISS) in survival prediction
Source: PLoS One. 2022 Oct 27;17(10):e0276624. doi: 10.1371/journal.pone.0276624 (PMC9612528; doi:10.1371/journal.pone.0276624)
Supplement: S7 Table — Patient counts reported for those in testing data. WHO: World Health Organization. (DOCX) [file pone.0276624.s007.docx]

| **Metric** | **Base model, 60:40 split**  **(n = 552,296)** |  | **Full model, 60:40 split**  **(n = 552,296)** |  | **WHO age≥75**  **(n = 135,665)** |  | **Base model, 50:50 split, with WHO age**  **(n = 690,370)** |  |
| --- | --- | --- | --- | --- | --- | --- | --- | --- |
|  |  |  |  |  |  |  |  |  |
| AUC | 0.941 (0.941-0.942) |  | 0.951 (0.951-0.951) |  | 0.850 (0.848-0.851) |  | 0.939 (0.939-0.939) |  |
| Recall | 0.996 (0.996-0.996) |  | 0.996 (0.996-0.996) |  | 0.993 (0.993-0.993) |  | 0.996 (0.996-0.996) |  |
| Precision | 0.982 (0.982-0.982) |  | 0.982 (0.982-0.983) |  | 0.966 (0.966-0.966) |  | 0.982 (0.982-0.982) |  |
| Specificity | 0.421 (0.419-0.423) |  | 0.439 (0.436-0.441) |  | 0.301 (0.298-0.305) |  | 0.413 (0.410-0.416) |  |
| Balanced Accuracy | 0.708 (0.707-0.709) |  | 0.717 (0.716-0.718) |  | 0.647 (0.646-0.649) |  | 0.704 (0.703-0.706) |  |
| Brier Score | 0.022 (0.022-0.022) |  | 0.021 (0.021-0.022) |  | 0.040 (0.040-0.040) |  | 0.022 (0.022-0.022) |  |
|  |  |  |  |  |  |  |  |  |

S7 Table. Additional sensitivity analyses of XGBoost models for parameters shown. Patient counts reported for those in testing data. WHO: World Health Organization
