# Supplementary material for: ICD-10 based machine learning models outperform the Trauma and Injury Severity Score (TRISS) in survival prediction
Source: PLoS One. 2022 Oct 27;17(10):e0276624. doi: 10.1371/journal.pone.0276624 (PMC9612528; doi:10.1371/journal.pone.0276624)
Supplement: S1 Fig — ISS: Injury Severity Score, ML: machine learning, RR: respiratory rate, SBP: systolic blood pressure, GCS: Glasgow Coma Scale. (DOCX) [file pone.0276624.s008.docx]

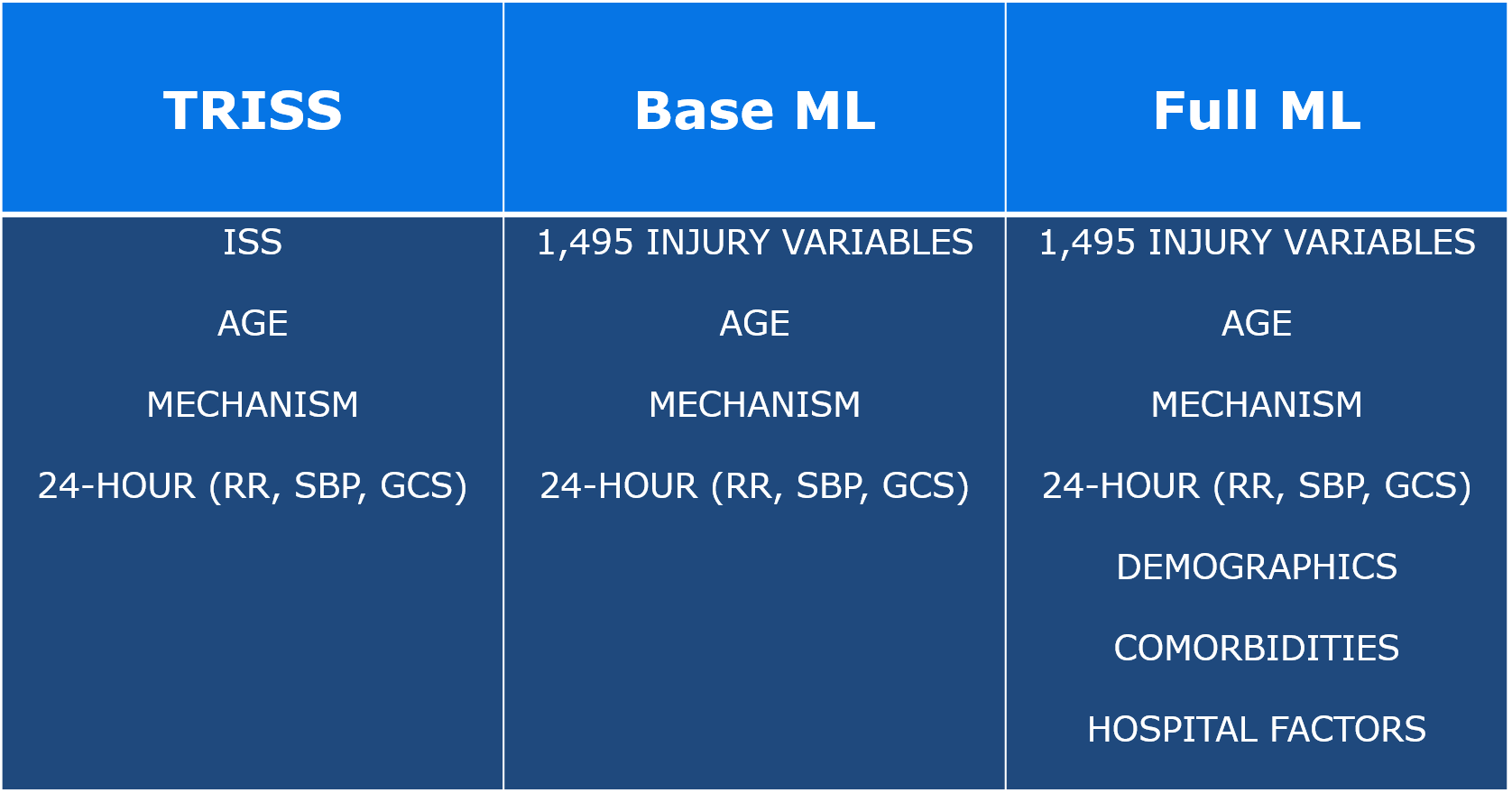


S1 Figure. Schematic demonstrating variables used in each model. ISS: Injury Severity Score, ML: machine learning, RR: respiratory rate, SBP: systolic blood pressure, GCS: Glasgow Coma Scale
