# Supplementary figures and images for: ICD-10 based machine learning models outperform the Trauma and Injury Severity Score (TRISS) in survival prediction
Source: PLoS One. 2022 Oct 27;17(10):e0276624. doi: 10.1371/journal.pone.0276624 (PMC9612528; doi:10.1371/journal.pone.0276624)

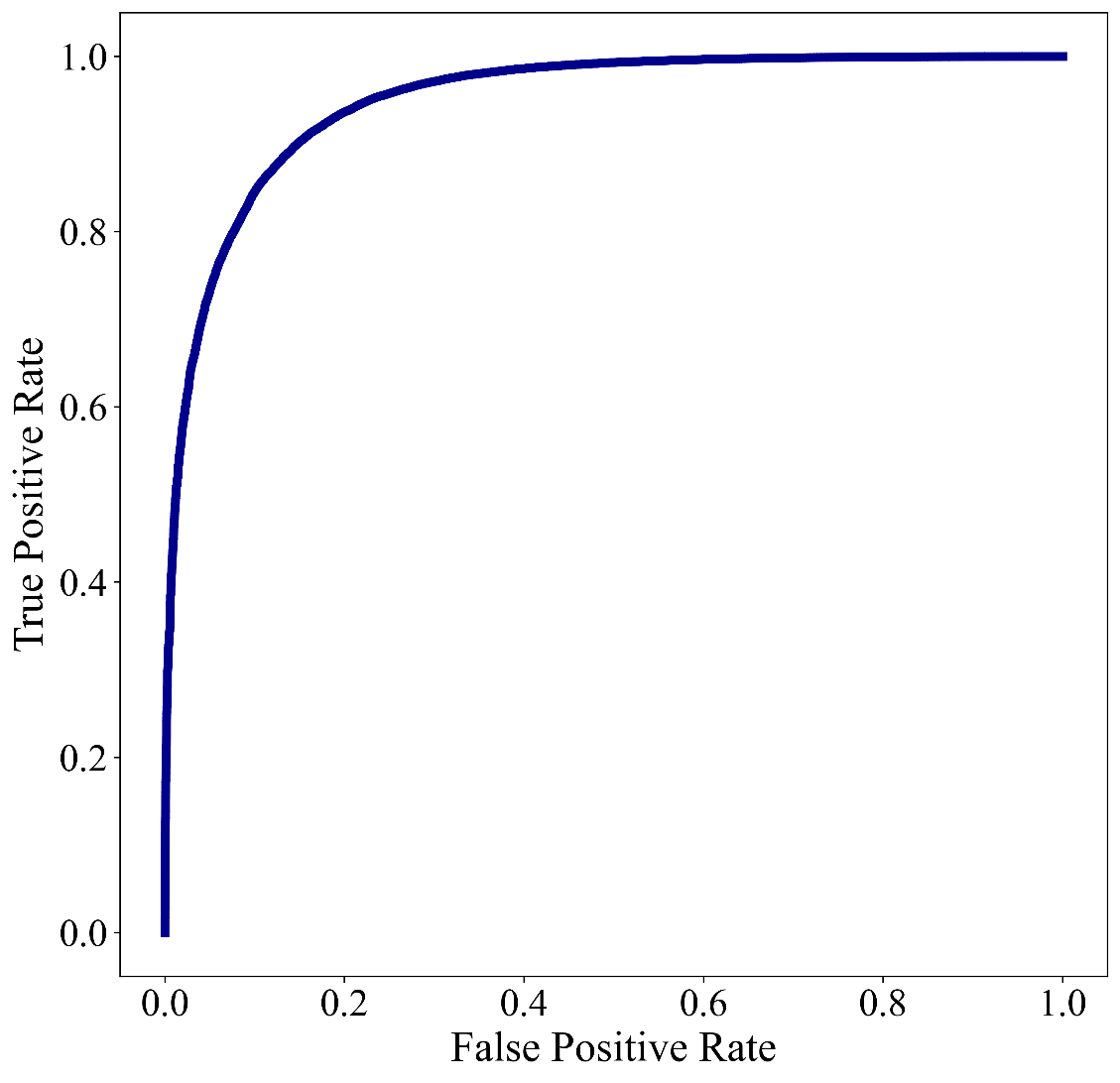

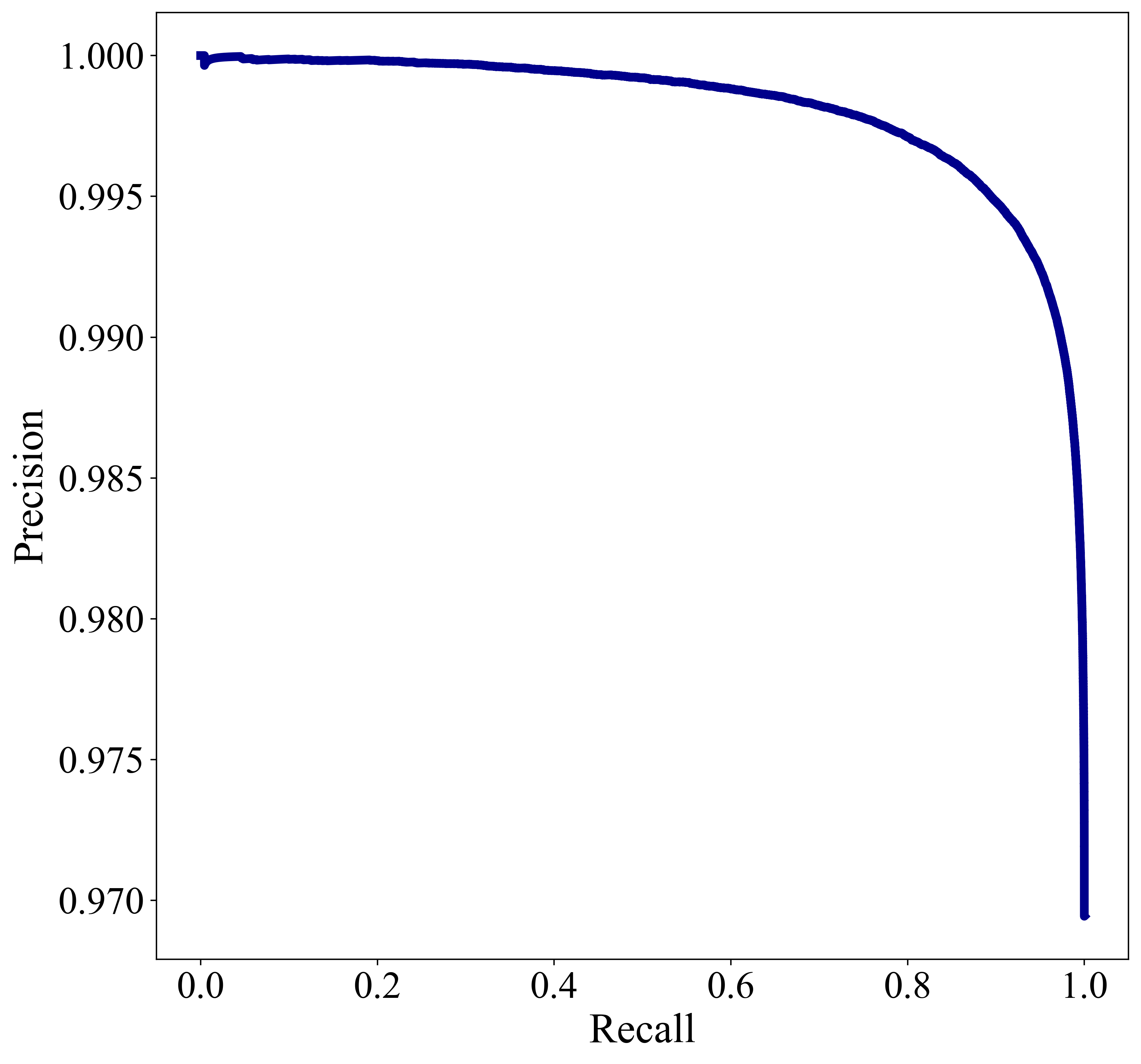


S2 Figure. Area under curve and precision-recall of full XGBoost model. AUC: 0.960.

Supplement: S2 Fig — AUC: 0.960. (DOCX) [file pone.0276624.s009.docx]

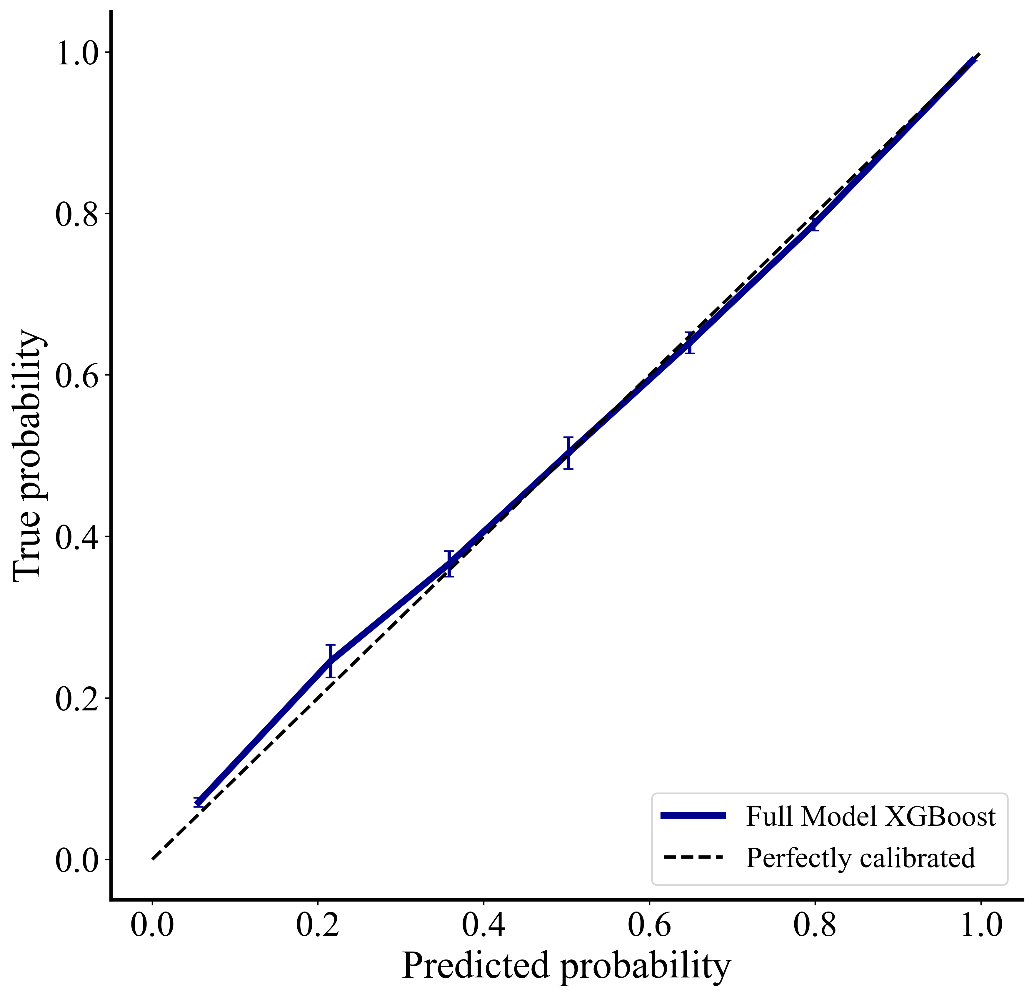


S3 Figure. Calibration curve of full XGBoost model. R2: 0.998.

Supplement: S3 Fig — R2: 0.998. (DOCX) [file pone.0276624.s010.docx]
